# Effectiveness of Low-Level Laser Therapy in Patients with Discogenic Lumbar Radiculopathy: A Double-Blind Randomized Controlled Trial

**DOI:** 10.1155/2022/6437523

**Published:** 2022-02-27

**Authors:** Ishaq Ahmed, Mohammad Ali Mohseni Bandpei, Syed Amir Gilani, Ashfaq Ahmad, Faryal Zaidi

**Affiliations:** ^1^University Institute of Physical Therapy, The University of Lahore, Islamabad, Pakistan; ^2^Pediatric Neurorehabilitation Research Center, University of Social Welfare and Rehabilitation Sciences, Tehran, Iran; ^3^Faculty of Allied Health Sciences, The University of Lahore, Lahore, Pakistan; ^4^University Institute and Clinics of Physical Therapy, The University of Lahore, Lahore, Pakistan

## Abstract

**Purpose:**

To determine the effectiveness of low-level laser therapy (LLLT) in patients with discogenic lumbar radiculopathy and correlation among pain intensity, functional disability, and lumbar range of motion (LROM). *Study Design/Setting*. A double-blind RCT was conducted at physical therapy departments of different hospitals of Islamabad, Pakistan. The study period was March 2020 to August 2021. *Patient Sample*. The study comprised 110 patients with acute LBP and unilateral discogenic lumbar radiculopathy. *Outcome Measures*. The outcomes of the treatment were measured on the first day and then after 18 sessions from each patient's pain intensity, functional disability, L-ROM, and straight leg raise by using visual analogue scale, Oswestry disability index, dual inclinometer, and straight leg raise test.

**Methods:**

A total of 110 participants with a mean age of 38 ± 7.4 years were randomly assigned into two groups of 55 each. The experimental group of 55 patients was treated with LLLT and conventional physical therapy. The control group of 55 patients was treated with conventional physical therapy alone. Both groups had received 18 treatment sessions. The data were analyzed through SPSS-21.0.

**Results:**

The results of the Wilcoxon signed-rank test score as well as Mann–Whitney U test indicated a statistically significant difference in values (*p* < 0.05 in all instances) within the groups and between the groups, respectively.

**Conclusions:**

The LLLT is proved as an efficient adjunct therapy to conventional physical therapy for discogenic lumbar radiculopathy.

## 1. Introduction

Lumbar disc prolapse is a movement of disc material, that is, nucleus pulposus or annulus fibrosis out of the intervertebral disc space. The ruptured nucleus comes in contact with the nearby nerves, causing their compression that in turn results in severe radicular pain [1–5]. Typically, the pain is located bilaterally at the posterior beltline with a sharp, shooting pain running down the low back, buttocks, and down the thigh along with numbness or tingling [6, 7]. The pain is aggravated by sitting, prolonged standing, bending, or twisting movements and is relieved by walking, lying down, resting, and lying down [7–10]. The history, symptoms, and physical examination of the patient are the tools for diagnosis. At some point in the evaluation, tests (X-ray, magnetic resonance imaging, or computed tomography scans) may be performed to confirm or rule out other causes of symptoms [1, 2, 11].

Lumbar disc prolapse is one of the major risk factors for low back pain [4, 9, 12–15]. About 90% of disc prolapse occurs in the lumbar region (L4-L5 or L5-S1) [9–15]. LBP due to disc prolapse is usually self-limited and of short duration [4]. The male-to-female ratio is approximately 1:1 [10–16].

The individuals aged between 25 and 55 years are mostly affected [1–5]. The risk factors for disc prolapse are age, disc trauma, degeneration, congenital predisposition, activity level, smoking, obesity, vibration (e.g., driving a car), and improper lifting technique, usually associated with bending with back rather knees, twisting violently, and so on [3, 5, 8, 16].

The prevalence of lumbar radiculopathy due to disc prolapse is somewhat lower, ranging from 17% to a little over 50% using validated instruments [9, 14, 17]. In two studies using strict criteria, the lifetime prevalence of radiculopathy due to a herniated lumbar disc was estimated to be 4% in females and 5% in males [10, 11]. The overall incidence of discogenic lumbar radiculopathy is 4.86 per 1,000 person-years.

The multiple treatment approaches are used for discogenic lumbar radiculopathy in order to decrease the intradiscal pressures, to increase fluid and nutrient exchange, to promote disc regeneration, and to retract nucleic material of bulging or herniated disc [14–18]. These approaches include strict bed rest, analgesics and anti-inflammatory drugs, muscle relaxants, electrotherapy, therapeutic exercises, soft tissue manipulation, tapping, lumbar corsets, laser therapy, or surgery as a last resort [8,9,19].

Laboratory studies show that low-level laser therapy (LLLT) modifies the inflammatory process and thus relieves the painful symptoms produced by disc lesions [15–21]. This modification is brought about by a decrease in nerve conduction, release of endogenous opioids, increase in angiogenesis, and consequently, increase in local microcirculation. It may also have inhibitory effects on the release of prostaglandins, cytokine levels, and cyclooxygenase (Cox), and it may accelerate cell proliferation, collagen synthesis, and tissue repair [15, 22–24].

In the reviewed literature, there seems to be a lack in establishing the effectiveness of LLLT when combined with conventional physical therapy. Discogenic lumbar radiculopathy is very common in our setup due to an increased workload, increased number of road traffic accidents, poor postures and socioeconomic status, deficient medical or rehab services, and so on. Thus, the current study was aimed to evaluate and generalize the comparative effects of conventional physical therapy combined with LLLT and conventional physical therapy alone in our current settings. Moreover, in the previous researches, there is a variation/gap regarding the dosage, wavelength, and duration of the application of LLLT. In this study, a different combination of these variables was applied. We hypothesize that LLLT combined with conventional physical therapy would provide a clinically and statistically significant benefit over a conventional physical therapy alone for patients with discogenic lumbar radiculopathy. At the same time, we aimed to suggest an appropriate and effective treatment proposal for patients with discogenic lumbar radiculopathy for LLLT, which is one of the new treatment options.

## 2. Methodology

### 2.1. Patients

A double-blind randomized controlled trial was conducted at physical therapy departments of three different hospitals, comprising data related to the 18-month period from March 2020 to August 2021. The study sample size was calculated using (formula: *n* = [z2 ∗*p* ∗ (1 – *p*)/e2]/[1 + (z2 ∗*p* ∗ (1 – *p*)/(e2 ∗N))], and a sample of 110 patients with acute low back pain and unilateral discogenic lumbar radiculopathy was obtained through nonprobability purposive sampling, who had symptoms for less than 4 weeks, confirmed by magnetic resonance imaging (MRI). A total of 110 participants (both males and females with a mean age of 38 ± 7.4 years) were randomly assigned into two equal groups of 55 (50%) each by sealed envelope method (see CONSORT flow chart). The experimental group of 55 patients (25 females and 30 males) with an average age of 37.24 + 7.414 years and control group of 55 patients (28 females and 27 males) with an average age of 39.00 + 7.49 years (Table 1) were selected in the study on the basis of unilateral leg pain greater than low back pain, leg pain below the knee to foot or toes follows a dermatomal pattern [7, 10], numbness and paresthesia in the same affected area, patients with moderate to severe score (21%–60%) in ODI [1,5,6], positive SLR/Lasegue's test (painful between 35 degrees and 75 degrees) [3, 4], and pain: limiting function and at least 3/10 on VAS, limited lumbar range of motion; flexion 25%, and extension 20%. Every patient was volunteered for his/her inclusion in the study with the criteria of no lumbar spine surgery within the past year, not receiving treatment by other methods for past 3 months and no spinal deformity and comorbid diseases. The trial was registered in ClinicalTrials.gov (IRCT20190707044128N2). Every patient had duly signed an informed consent that was approved by the Institutional Review Board/Ethical Committee; therefore, the rights of every patient were protected.


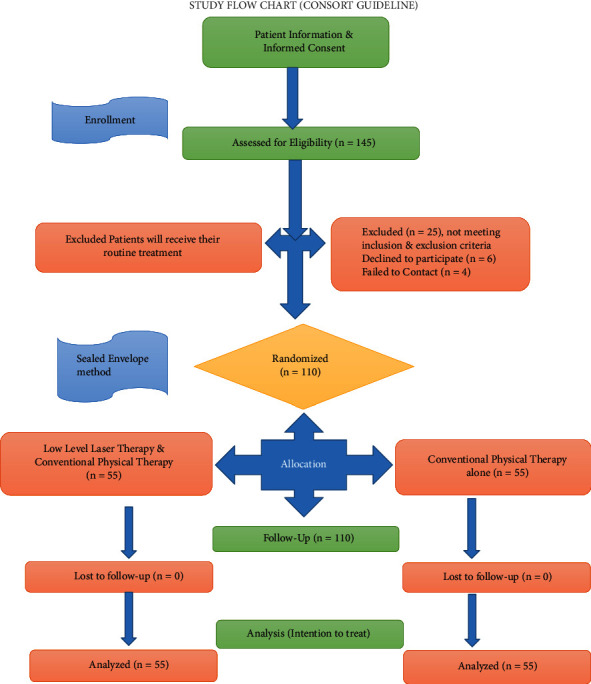


### 2.2. Blinding Scheme

One hundred and ten (110) sequentially numbered sealed envelopes were prepared by the researcher. The content of envelopes stated which treatment protocol was to be used without mentioning the groups. The patients and the assessor were kept blind about the allocation.

### 2.3. Treatment Procedure

Patients once accepted into the study were randomly assigned to a treatment group by sealed envelope method, either an experimental group (low-level laser therapy (LLLT) – 830 nm, 0.67 W/cm^2^ or 300 mW/cm^2^, and conventional physical therapy–back extension exercises, hot pack, hold relax with sustained stretches in SLR, and sciatic nerve mobilizations) or a control group (conventional physical therapy alone). Each patient therefore had an equal chance of being selected in either group. The laser probe was held stationary in skin contact with special care of skin hygiene at 2.5 and 3.5 cm laterally of the spinous process of the involved nerve root (L4 or L5 or S1) and one distal level segment [17]. The parameters [14] of the low-level laser beams are presented in Table 2. The hot pack was placed on the lower lumbar region (L3-S2) for 10 minutes [5, 7]. During back extension exercises, patients were asked to perform lumbar extension exercises in prone lying position in 3 sets of 5 repetitions each [1, 5]. Hold relax technique was applied for hamstrings, gluteus maximus, and calf muscles followed by a sustained stretch in 3 sets of 5 repetitions each with 15 seconds hold [25–27]. Sciatic nerve mobilizations were performed 5 times in one set with 15 seconds hold [28, 29].

The patients in both groups had received a series of 18 treatment sessions (3 sessions per week for 6 to 8 weeks). The treatment days for the experimental and control groups were alternate in nature.

### 2.4. Measurable Outcome Variables

The outcomes of the treatment were measured on the first day and then after the 18 sessions from each patient's pain intensity, functional disability, lumbar range of motion (L-Flexion and L-Extension) and straight leg raise. VAS was used for measuring the pain intensity in affected leg [8]. It consisted of a horizontal scale graded from 0, representing no pain, to 10, representing the worst imaginable pain. A dual inclinometer was used for measuring the lumbar range of motion (L-ROM) in which PSIS to 15 cm cephalad landmarking technique was used. The upper and lower spinal landmarks were marked by a horizontal line on a piece of adhesive tape. The adhesive marks were removed and relandmarked for each set of data. The two heads of the dual inclinometer were placed at the low marked levels along the spine; the MASTER head was placed at the upper landmark and the SLAVE head at the lower landmark. The patient was instructed to bend forward maximally, while changes in degrees were recorded. The intrarater reliability of this method using a dual inclinometer is 0.73 for L-Flexion and 0.85 for L-Extension [15]. ODI (Oswestry disability index) is used for measuring functional disability. It consists of 10 questionnaires about how pain affects daily activities, scored from 0 to 5 for each section, with higher values indicating a more severe impact. The Cronbach *α* reliability score for ODI was 0.877 [15]. Straight leg raise (SLR) includes hip flexion by keeping the knee extended. A pooled sensitivity for the straight leg raising test is 0.91 (95% CI 0.82–0.94), and a pooled specificity is 0.26 (95% CI 0.16–0.38) [8]. The test is based on stretching of the nerves in the spine [9].

### 2.5. Data Analysis

The analyses were done on the basis of intention to treat using SPSS version 21.0 for statistical analyses. The results were expressed as median (25% and 75% percentiles) as data were not normally distributed measured through Shapiro–Wilk test (*p* value < 0.05, CI 95%). Mann–Whitney U test for intergroup analysis and Wilcoxon signed-rank test for intragroup analysis were used as tests of significance, and two-tailed *p* value of 0.05 (CI 95%) was calculated to either accept or reject the null hypothesis. Effect size (Cohen's d) analysis was done for evaluating the importance of measured changes in pain intensity, disability, L-ROM, and straight leg raise, as post hoc power analyses. Correlation coefficient/Pearson's correlation was used to determine the inter- and intragroup correlation among pain intensity, L-ROM, and functional disability.

## 3. Results

### 3.1. Intergroup Statistical Analysis

**Figure 1:**
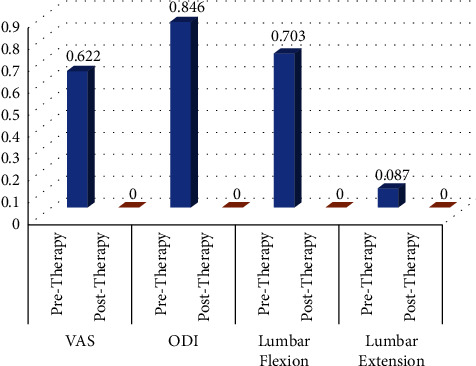
Intergroup analysis of measured changes between the groups.

The results from intergroup statistical analysis measured through the Mann–Whitney U test showed no statistically significant differences between the groups at baseline characteristics (*p* > 0.05 in all instances), while statistically significant differences were found in posttreatment values between the groups (*p* < 0.05 in all instances). The results from the VAS, ODI, and dual inclinometer statistical analysis at the end of treatment revealed that there was statistically significant differences in values present between experimental and control groups over time. This indicates that both the experimental and control groups had shown a significant improvement in their individual outcomes at specific intervals of time. However, based on effect size calculations the importance of measured differences at the end of treatment to be large for pain intensity, functional disability, and lumbar flexion and extension (*d* = 0.984 to 3.641). The overall effect size was highest for lumbar extension and least for lumbar flexion between the groups (shown in Table 3 and Figure 1).

**Figure 2:**
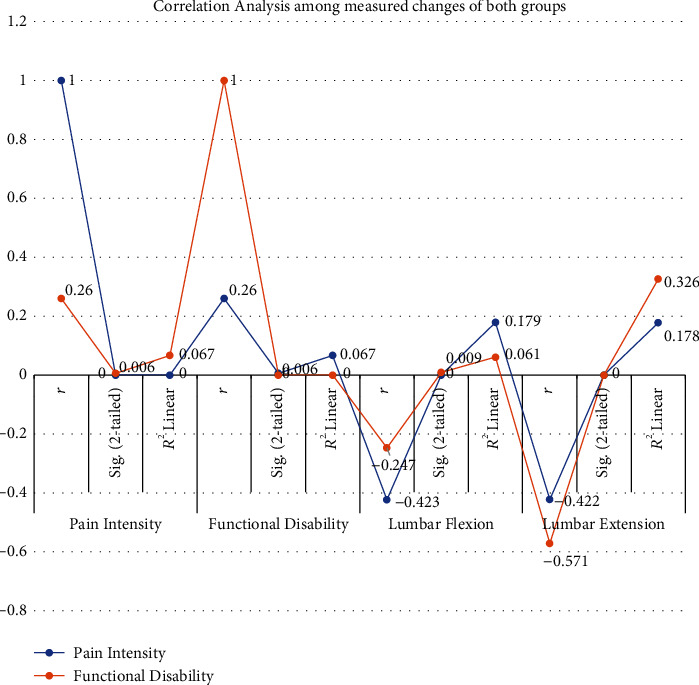
Correlation analysis among measured changes of both groups.

The analysis of correlation (shown in Table 4 and Figure 2) showed that the strength of correlation between variables was weak to moderate (*r* = 0.24 to 0.57) with statistically significant correlation coefficient (*p* < 0.05). It is concluded that only 7% (*r*2 = 0.067) of the variation in functional disability, 18% (*r*2 = 0.179) in lumbar flexion, and 18% (*r*2 = 0.178) in lumbar extension are explained by pain intensity. However, only 6% (*r*2 = 0.061) of the variation in lumbar flexion and 32% (*r*2 = 0.326) in lumbar extension is explained by functional disability.

### 3.2. Intragroup Statistical Analysis

The results from intragroup statistical analysis showed a statistically significant improvement within the groups (*p* < 0.05 in all instances). The median values with 25% and 75% percentiles for both groups (experimental and control groups) had shown significant differences in values with respect to pain intensity, functional disability, and lumbar flexion and extension that were obtained over time. Hence, both the groups had shown clinical improvements that were statistically significant. These statistically significant changes on each measured outcome through the Wilcoxon signed-rank test were consistent throughout the treatment session as measured at first and last visits for both the groups. Effect size statistical analysis for the experimental group had shown the importance of measured differences to be large for pain intensity, functional disability, and lumbar flexion and extension (*d* = 2.51 to 3.49), while for the control group, it was large for pain intensity, functional disability, lumbar flexion, and medium for lumbar extension (*d* = 0.69 to 3.69). The overall effect size was highest for pain intensity and least for lumbar extension for both groups.

**Figure 3:**
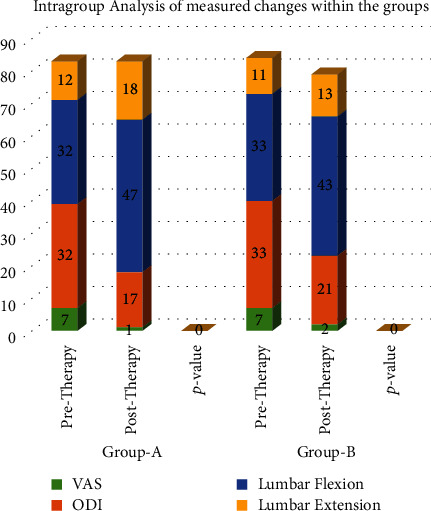
Intergroup analysis of measured changes within the groups.

It is evident from the median values with 25% and 75% percentiles of measured outcomes (shown in Table 5 and Figure 3) that the experimental group had produced more improvement in all quantitative variables than that of control group. Moreover, the percentage of negative SLR at the end of treatment was also high (98.2%) in the experimental group than that of the control group (91%; shown in Table 1).

**Figure 4:**
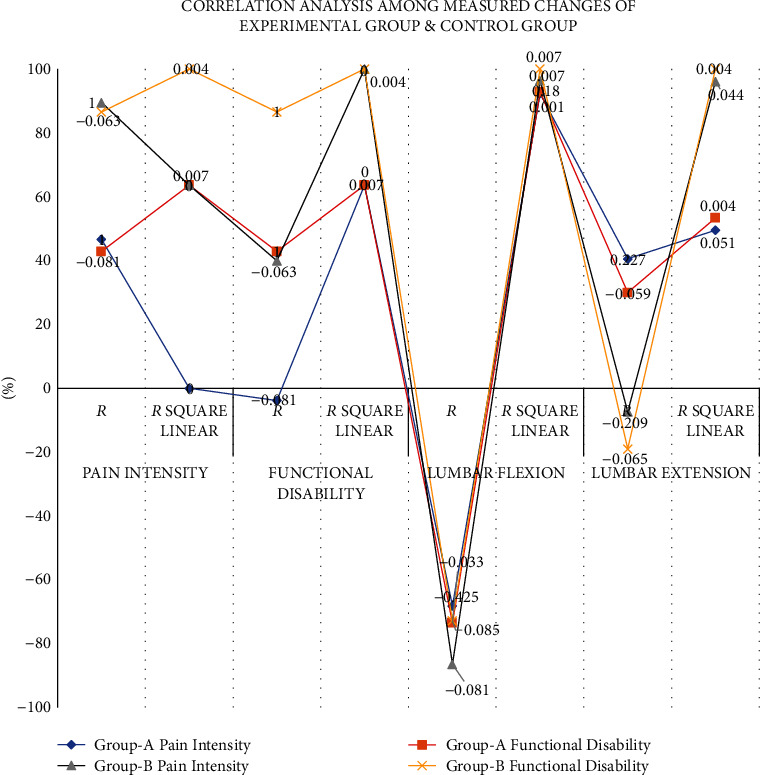
Correlation analysis among measured changes experimental and control groups.

The analysis of correlation for the experimental group (shown in Table 6 and Figure 4) showed that the strength of correlation between variables was weak to moderate (*r* = 0.033 to 0.425) with statistically Insignificant correlation coefficient (*p* > 0.05) except for lumbar flexion (*p* < 0.05). It is concluded that only 0.7% (*r*2 = 0.007) of the variation in functional disability; 18% (*r*2 = 0.18) in lumbar flexion, and only 5% (*r*2 = 0.051) in lumbar extension are explained by pain intensity. However, only 0.1% (*r*2 = 0.001) of the variation in lumbar flexion and 0.4% (*r*2 = 0.004) in lumbar extension are explained by functional disability.

The analysis of correlation for the control group (shown in Table 4 and Figure 4) showed that the strength of correlation between variables was weak (*r* = 0.063 to 0.209) with statistically insignificant correlation coefficient (*p* > 0.05). It is concluded that only 0.4% (*r*2 = 0.004) of the variation in functional disability, 0.7% (*r*2 = 0.007) in lumbar flexion, and only 4% (*r*2 = 0.044) in lumbar extension are explained by pain intensity. However, only 0.7% (*r*2 = 0.007) of the variation in lumbar flexion and 0.4% (*r*2 = 0.004) in lumbar extension are explained by functional disability.

## 4. Discussion

### 4.1. Principal Findings

In the current study, we had extrapolated the effects of conventional physical therapy with and without low-level laser therapy on the pain intensity, functional disability, lumbar ROM, and straight leg raise in patients with discogenic lumbar radiculopathy. The changes that were found in this study from 0 day to 45th day of intervention were remarkable. The age range for the study was 25–55 years with similar mean age of 38.12 + 7.14 (Table 2) reported for each group. The age restriction of 25–55 years is due to a reason that discogenic lumbar radiculopathy is more evident in this range [1–5]. Kreiner et al. also utilized an age restriction of 25–55 years for the same reasons [1]. Freburger JK et al. reported an age range of 25−40 years in 50% of the participants in their study with an average age of 34 years [4]. The distribution of men and women (Table 2) among the two groups were almost similar; however, men comprised 52% of the participants in the study. It is due to the high prevalence of lumbar radiculopathy is estimated to be 2–5% in men and 1–3% in women [4, 6, 9]. The BMI scores (Table 1) between the two groups were almost similar, that is, 26.9 ± 3.65 with *p* value = 0.878.

The intragroup as well as intergroup analyses showed significant changes in improving pain intensity, disability level, lumbar ROM, and straight leg raise in both groups, that is, experimental and control groups, which means that both the groups had produced individual results that were different from each other. It is evident from the mean and standard deviations of intragroup differences of outcomes that the experimental group had shown better results than that of the control group. The most prominent are the results on reduction in functional disability (from moderate to minimal disability) and improvement in lumbar flexion range (28%; accounted for as a difference between percentage of pre- and post-mean values). However, the pain intensity was reduced (severe to moderate level) as well with large effect size between the groups. The improvement in the lumbar extension range was not that much significant for both groups, but here also the experimental group shows better results than that of the control group with a large effect size in the experimental group and medium size in the control group.

Moreover, the percentage of negative SLR/Laseague's test at the end of treatment was high (98.2%) in the experimental group than that of the control group (91%). SLR is used as the first test to detect disc hernia, and it is of 70–80% accuracy [13]. Specificity rate of the SLR test was 89% [13].

On analysis of intragroup correlation, experimental group showed weak to moderate strength of correlation between outcome variables where large negative linear correlation (18%) found between pain intensity and lumbar flexion and least (0.7%) with functional disability, while there were almost weak or no linear correlation (0.1 to 0.4%) between functional disability and lumbar ROM. The control group showed a weak linear correlation between outcome variables, comparatively high (4%) between pain intensity and lumbar extension. The correlation analysis between outcome variables of both groups showed that the strength of correlation was weak to moderate with a statistically significant correlation coefficient (- < 0.05), where a large negative linear relationship was found between pain intensity and lumbar ROM (18%) and (32%) between functional disability and lumbar extension. It means that all the variables are interrelated to each other, and any change in one variable brings about a change in another variable either in positive direction or negative/inverse direction.

### 4.2. Comparison of the Results to Other Studies

The major problems in comparing the results of this study with others are the differences in the included patients and applied laser specifications. A meta-analysis by Glazov et al. considered nonspecific LBP having no consistent conclusions [24]. In addition, many other clinical studies have used LLLT for nonspecific chronic LBP; however, the patient population was very heterogenic, and t their pain production was not only caused by the pathological changes in the spinal and paraspinal structures but also caused by complex neurophysiologic and psychosocial mechanisms. Studies by Yousefi-Nooraie et al. compared the effects of LLLT with therapeutic exercises in chronic LBP [22].

Doğan et al. used LLLT doses through the source of laser beams NdYAG36 recommended by WALT in a group of patients with nonspecific LBP [20]. Milica Jovi et al. conducted a study with the idea of getting additional anti-inflammatory effects in a group of patients with acute LBP [15]. However, the results were difficult to compare in these studies due to the heterogeneity of patients in their pathophysiological aspect. In a study to see the effects of different therapies in patients with acute LBP with radiculopathy, an 830 nm laser unit at a dose of 1 J was used. There were no significant changes in results obtained compared to ultrasound and traction therapy [17].

There are multiple biological actions of LLLT that include a direct stimulating effect on nerve fibers ensuring rapid recovery from conduction block, a pronounced decrease in inflammatory response as a primary effect as well as an improvement in neurophysiologic features of nerve structures, and a remarkable changing effects in the endorphin level. Out of them, the most evident are the anti-inflammatory effects documented in many experimental studies. The studies involving experimentally induced inflammation methods have recorded various changes in biochemical markers of inflammation, cellular chemotaxis, decrease in the process of oedema formation, haemorrhage, and necrosis by using the local laser with different laser beams at 660 nm, 684 nm [15], 780 nm [23], and 904 nm [24], respectively. This mode of reduction is in positive correlation with a decrease in TNFa level and is dose-dependent [24]. The direct impact of LLLT on neural structures is evident in acute lesions, such as acute lumbar radiculopathy that leads to an onset of neuropathic pain. The use of laser therapy at 840 nm wavelength, on injured peripheral nerves, significantly improves nerve recovery in clinical studies [17]. The impact of LLLT on the activity of anti-oxidative enzymes played a role in the modulation mechanism as these enzymes increase the nonspecific resistance of cells to different damages [21–23]. The possibility of some positive interactions between LLLT and COX-2 inhibitors should be considered [23].

The lack of evidence with regard to diagnostic procedures and treatment interventions for a condition that occurs as frequently as degenerative lumbar radiculopathy is very distressing. The main characteristics of published trials are imprecise selection of patients with lumbar radiculopathy nonconfirmed with additional MRI and EMG investigations, with different clinical characteristics, undefined clinical stage, and usually a lack of description of treatment.

### 4.3. Strengths and Limitations

The strength of this study is our sample. We recruited from a variety of surgeons making our results generalizable. The results of this study must be considered in light of several limitations. Patients with relatively strictly defined clinical forms of the condition (severe levels of pain and moderate-severe levels of disability) were selected due to the typical flow of patients to clinical treatment (selection bias). Randomization did not include the initial level of disability, MRI and EMG findings, duration of symptoms, or other psychosocial characteristics that could influence the therapeutic response. The results of this study suggest only short-term effects. The identification of true positive effects under conditions of this study is controversial given that we had no untreated group, especially when the history, level, and percentage of spontaneous recovery were unknown.

### 4.4. Recommendations

Future studies could include patients that are randomized by levels for baseline disability, duration of symptoms, and other physical and psychosocial characteristics that could influence the response to treatment. In addition, further long-term studies could be designed that compare the use of a single type of therapy with a combined therapy approach. Further understanding of the mechanisms of the effects of LLLT could be very important for clinical recommendations with respect to the laser parameters, the area of irradiation, and duration of treatment.

## 5. Conclusions

The low-level laser therapy at a wavelength of 830 nm and a dose of 3 J/point for the acute LBP with discogenic lumbar radiculopathy, proved as an efficient adjunct therapy to conventional physical therapy in significantly improving local trunk movements, pain intensity, and related functional disability, as compared to conventional physical therapy alone. Moreover, the strength of correlation among the variables of the experimental group is more pronounced than that of control group. In addition, no major side effects were observed during and after the use of LLLT.

## Figures and Tables

**Table 1:** Basic characteristics of patients in groups.

Groups	Age	BMI (kg/m^2^)	Gender	Pretest straight leg raise		Posttest straight leg raise	
	Mean + SD	Mean + SD (*p*-value)	Male:female	Positive (*n*)	Negative (*n*)	Positive (*n*)	Negative (*n*)
Group A	37.24 + 7.414	26.9 ± 3.65 (0.878)	30:25	55 (100%)	0%	1 (1.8%)	54 (98.2%)
Group B	39.00 + 7.49	26.7 ± 3.65 (0.878)	27:28	55 (100%)	0%	5 (9%)	50 (91%)

**Table 2:** Characteristics of laser beams.

Wavelength	830 nm (near infrared)
Laser frequency	5,000
Power output	100 mW
Power density	300 mW/cm^2^ or 0.67 W/cm^2^
Energy	3 J/point
Energy density	3 J/cm^2^ on each point
Number of points	4
Spot size	1 cm
Treatment time	30 sec on each point
Daily energy delivered	12 J

**Table 3:** Statistical analyses of measured changes between the groups.

	VAS		ODI		Lumbar flexion		Lumbar extension	
	Pretherapy	Posttherapy	Pretherapy	Posttherapy	Pretherapy	Posttherapy	Pretherapy	Posttherapy
Mann–Whitney U test	1.43 *E* + 03	710	1.48 *E* + 03	341	1449	736.5	1228.5	0
Z-score	−0.494	−4.959	−0.195	−7.045	−0.381	−4.663	−1.714	−9.086
Asymp. sig. (two-tailed)	0.622	0.000	0.846	0.000	0.703	0.000	0.087	0.000
Effect size (*d*^*∗*^)	−1.104		−1.588		0.984		3.641	

*d* ^*∗*^: Cohen effect size: *d* < 0.2, small; 0.2 < *d* < 0.8, medium; and *d* > 0.8, large.

**Table 4:** Correlation analysis among pain intensity, functional disability, and lumbar ROM outcomes of both groups.

Both groups	Pain intensity			Functional disability			Lumbar flexion			Lumbar extension		
Outcomes	*r*	Sig. (two-tailed)	*R*-squared linear	*r*	Sig. (two-tailed)	*R*-squared linear	*r*	Sig. (two-tailed)	*R*-squared linear	*r*	Sig. (two-tailed)	*R*-squared linear
Pain intensity	1			0.26	0.006	0.067	−0.423	0.000	0.179	−0.422	0.00	0.178
Functional disability	0.26	0.006	0.067	1			−0.247	0.009	0.061	−0.571	0.00	0.326

Pearson correlation coefficient: *r* < 0.4, weak or no relationship; 0.5 < *r* < 0.7, moderate relationship; and *r* > 0.7, strong relationship.

**Table 5:** Median values (25% and 75% percentiles) and paired sample statistics of measured outcomes.

Outcomes	Group A		Wilcoxon signed-rank test score		Effect size	Group B		Wilcoxon signed-rank test score		Effect size
	Pretherapy	Posttherapy	Z-score	Asymp. sig. (two-tailed)	*d* ^*∗*^	Pretherapy	Posttherapy	Z-score	Asymp. sig. (two-tailed)	*d* ^*∗*^
VAS	7 (6; 8)	1 (0; 2)	−6.457	0.000	3.49	7 (6; 8)	2 (2; 3)	−6.515	0.000	3.69
ODI	32 (29; 35)	17 (16; 19)	−6.457	0.000	3.39	33 (28; 37)	21 (20; 25)	−6.462	0.000	3.14
LFLEX^∗^	32 (29; 36)	47 (44; 49)	−6.461	0.000	3.11	33 (30; 37)	43 (42; 45)	−6.456	0.000	2.52
LEXT^∗^	12 (9; 13)	18 (17; 20)	−6.46	0.000	2.51	11 (8; 13)	13 (11; 14)	−4.166	0.000	0.69

d^*∗*^: Ccohen effect size: *d* < 0.2, small; 0.2 < *d* < 0.8, medium; and *d* > 0.8, large. LFLEX^*∗*^ = lumbar flexion range of motion. LEXT^*∗*^ = lumbar extension range of motion.

**Table 6:** Correlation analysis among pain intensity, functional disability and ROM outcomes of experimental and control groups.

		Pain intensity			Functional disability			Lumbar flexion			Lumbar extension		
Groups	Outcomes	*r*	Sig. (two-tailed)	*R*-squared linear	*r*	Sig. (two-tailed)	*R*-squared linear	*r*	Sig (two-tailed)	*R*-squared linear	*r*	Sig. (two-tailed)	*R*-squared linear
Group A	Pain intensity	1			−0.081	0.55	0.007	−0.425	0.00	0.18	0.227	0.09	0.051
	Functional disability	−0.081	0.55	0.007	1			−0.033	0.80	0.001	−0.059	0.66	0.004
Group B	Pain intensity	1			-0.063	0.64	0.004	–0.081	0.55	0.007	–0.209	0.12	0.044
	Functional disability	–0.063	0.64	0.004	1			0.085	0.53	0.007	–0.065	0.63	0.004

Pearson correlation coefficient: *r* < 0.4, weak or no relationship; 0.5 < *r* < 0.7, moderate relationship; and *r* > 0.7, strong relationship.

## Data Availability

The datasets used and/or analyzed during the current study will be available from the corresponding author on reasonable request.

## Abbreviations

LBP: Low back pain
LLLT: Low-level laser therapy
VAS: Visual analogue scale
ODI: Oswestry disability index
L-ROM: Lumbar range of motion
MRI: Magnetic resonance imaging
SLR: Straight leg raise.

## Additional Points

The findings of this study have several clinical implications for patients with discogenic lumbar radiculopathy as LLLT along with physical therapy is a noninvasive modality that can improve pain, functional disability, and L-ROM without disturbing the daily routine of the patients. Therefore, this is approved to be an effective treatment protocol to be used by physiotherapists for the treatment of discogenic lumbar radiculopathy.
